# Child marriage practices among the Rohingya in Bangladesh

**DOI:** 10.1186/s13031-020-00274-0

**Published:** 2020-05-25

**Authors:** Andrea J. Melnikas, Sigma Ainul, Iqbal Ehsan, Eashita Haque, Sajeda Amin

**Affiliations:** 1 Population Council, 1 Dag Hammarskjold Plaza, 3rd floor, New York, NY 10017 USA; 2 Population Council, Dhaka, Bangladesh

**Keywords:** Rohingya, Child marriage, Qualitative

## Abstract

**Background:**

Previous research suggests that child marriage may be accelerated during times of crisis and insecurity as resources are scarce and child marriage may be a survival strategy for girls and their families. In 2017, the Rohingya experienced a mass displacement to Bangladesh in response to escalating violence in Myanmar. This displacement has resulted in an estimated population of nearly 1 million Rohingya living in Cox’s Bazar.

**Methods:**

We conducted in-depth interviews (*n* = 48) and focus group discussions (*n* = 12) with Rohingya male and female adolescents and young adults (14–24 years), and program managers and service providers (*n* = 24) working in Cox’s Bazar to understand their experience of living or working in the camps, preferences for timing of marriage, and marriage practices in Myanmar and in the camps. We also interviewed Bangladeshis in the host community to complement our understanding of marriage in the camps and its influence in the broader community. Our primary objective was to describe how displacement influenced marriage timing and practices.

**Results:**

We found that child marriage is a strong cultural phenomenon among the Rohingya, primarily rooted in socio-cultural and religious beliefs around readiness for marriage. Although child marriage was practiced by the Rohingya in Myanmar, specific state law and oppression by military forces prevented many from marrying before age 18. Now this preference is more easily practiced in the camps in Bangladesh where the displaced Rohingya experience less marriage regulation. Host community participants perceive the presence of the Rohingya as encouraging both polygamy and child marriage in their communities, leading to tension among the host community.

**Conclusions:**

Our findings support evidence that conflict and displacement increase child marriage in populations already vulnerable to child marriage by exacerbating gender inequities. However, our findings also suggest group norms around religious and cultural preferences for age at marriage play a significant role in post-displacement behaviors surrounding marriage.

## Background

Child marriage, or marriage before age 18, is a significant problem faced by millions of girls each year. Although child marriage is often practiced to protect the physical security of girls [18], marriage at young ages can pose multiple threats to young girls’ lives, health, and future prospects [47, 53]. For example, child marriage is associated with a number of negative outcomes for girls including an increased risk of intimate partner violence, higher maternal mortality, and lower education levels [14, 21, 33, 38, 39]. Drivers of child marriage are context-specific, but most research suggests gender inequities including low status of women and girls, religious norms that dictate timing of marriage, importance of motherhood, and limited alternatives are important drivers of child marriage [15, 25, 44].

Despite global gains in addressing child marriage, including a decline from 25 to 21% of young women married under age 18 in the last decade [1], research suggests that there may be significant within-country variation due in part to contextual factors that accelerate vulnerability for girls. These include conflict, displacement, and food insecurity [15]; countries with high child marriage also tend to be fragile states [13]. Previous research in other humanitarian and post-conflict settings suggest that gender inequities may be accelerated during crises [4, 35] and following displacement [51]. It is also understood that social and economic factors that contribute to child marriage may be exacerbated by conflict and insecurity and may lead to increases in child marriage [35]. Refugee crises are characterized by a loss of livelihoods, decreased economic opportunity, heightened insecurity, and the absence of education, all of which can contribute to changes in marriage practices and increases in child marriage [17, 35]**.**

The Rohingya are a Muslim ethnic minority group that have been forcibly displaced due to increasing violence in the northern Rakhine state of Myanmar. According to the Myanmar Citizenship Law of 1982, Rohingya Muslims are considered to be “stateless” and “illegal immigrants” [49]**.** The process of “othering,” exclusion, discriminatory treatment, and “ethnic cleansing” of the Rohingya people in Myanmar is well documented [27, 30, 32, 42]**.** The Rohingya people have faced an extended period of severe and systemic oppression characterized by a lack of freedom of movement, limited access to sufficient food, inadequate health care, and restricted educational and livelihoods opportunities [40, 41]**.**

In August 2017, over 720,000 Rohingya migrated to Bangladesh creating the fastest growing refugee crisis in the world. Over a million displaced Rohingya now reside in Rohingya camps located in two upazilas, or administrative sub-districts, (Ukhia and Teknaf) of Cox’s Bazar, Bangladesh. The vast majority are women and children [22–24, 46] and almost 60% of the population are under the age of 18. The refugee camps are confined to an area of 5800 acres densely populated with nearly 1 million displaced persons [28, 50]. Water, sanitation, and environmental degradation are concerns for both the camps and the host community [28]. Although some Rohingya refugees have settled outside the camps, the majority have remained in the camps, which function as contained communities with services provided by the numerous aid groups working there.

The Rohingya’s displacement from Myanmar to Bangladesh includes moving from a situation of limited mobility and lack of citizenship within Myanmar due to government oppression to limited mobility and statelessness due to status as displaced persons residing within Bangladesh. While understanding the parallels of oppression in Myanmar versus Bangladesh is of interest, there are limited sources of information about life in the Rohingya camps in Bangladesh. Based on available reports from UN agencies and NGOs, we know that the Rohingya in Bangladesh are not allowed to pursue livelihood opportunities, and living conditions in the camps are congested and difficult [2]. In Bangladesh, a number of laws and administrative circulars also limit the ability of the Rohingya to officially register marriages and limit marriages between Rohingya and Bangladeshis [48].

Southern Bangladesh is highly vulnerable to climate change that can have devastating effects on temperature, flooding, erosion, and increased water and food insecurity [4, 12]. The Rohingya camps in Bangladesh are located in areas vulnerable to climate change. These vulnerabilities have now been exacerbated in the camps due to deforestation and population density [36]; recently the Rohingya camps have also been affected by landslides due to heavy rain [9]. As displacement and climate change both contribute to increased vulnerability to child marriage, the Rohingya refugee population in Bangladesh is at increased risk of child marriage.

There is limited ethnographic research on marriage practices among the Rohingya. Previous research from this project found similarities with other South-Asian Muslim communities including Bangladesh: marriages among the Rohingya are characterized by the practice of dowry, or the transfer of wealth from the bride’s family to the groom’s family at marriage, and sexual activity before marriage is discouraged [3]. Research on marriage and the Rohingya has largely focused on imposed marital restrictions due to their social position in Myanmar. Historically, institutionalized discrimination against Rohingya minorities in Myanmar included placing restrictions on marriage and birth registration [20]. Rohingya marriages in Myanmar required registration through military authorities and included identity checks and a large sum of money [30]. The Nasaka (abbreviation for “Nay-Sat Kut-kwey ye”) or the Myanmar border security force used to enforce Burmese laws in Rakhine state until 2013 enforced marriage laws, restricted mobility, and enforced restrictions on family size [27].

One common strategy to address child marriage across contexts is to strengthen and enforce age at marriage laws. Internationally, Article 16 of the Universal Declaration of Human Rights states that marriage shall be entered into “only with the free and full consent of the intending spouses” and by “men and women of full age” and the Convention on the Elimination of Forms of Discrimination Against Women declares child marriage illegal. In response to that, many countries have strengthened laws against marriage under age 18 [31]. Studies on marriage laws and their effects on behaviors have found that behaviors may be influenced by the existence of such laws: Ambrus et al. [5] found that levels of dowry and prenuptial agreements increased in response to legal barriers to polygamy and the increase in cost of divorce to men [5]. Cammack et al. [11] found that in Indonesia, laws did not appear to directly affect the trend in age at marriage. Rather, they suggest that values of choice and autonomy that were emphasized by the law better explain increasing age at marriage [11]. Research on laws related to age of marriage suggests that norms play an important role: perceptions of what others are doing and changing group norms may influence timing of marriage [8].

How does a displaced population facing risks for child marriage including insecurity and climate vulnerability, reckon with the changing enforcement of age at marriage laws? There is limited research on the effects of loosening laws related to timing of marriage; it is much more common for individuals to experience a tightening of restrictions on the practice of child marriage than a relaxing of those restrictions. The Rohingya in the camps present a unique example of relaxing restrictions combined with displacement and present an opportunity to understand how marriage practices may change in the face of such upheaval. The Rohingya displacement to Bangladesh also meant moving from Myanmar, a predominantly Buddhist country, to a Muslim country, and moving from a country with lower overall rates of child marriage (16% of women married before age 18 in Myanmar) [34] to a country with higher rates (59% of women married before age 18 in Bangladesh )[37]. In this paper we sought to:

- Understand how age at marriage and marriage practices changed among the Rohingya after displacement to Bangladesh; and
- Understand the local community’s perception of such practices and how the presence of the Rohingya may influence marriage practices in the host community.

## Methods

Data were collected as part of a larger project to examine marriage and sexual and reproductive health of the Rohingya in Bangladesh more broadly [3]. We conducted in-depth interviews and focus group discussions to better understand marriage practices among the Rohingya living in Cox’s Bazar. The study population included Rohingya male and female adolescents/young adults (ages 14–24), program managers and service providers working in the camps (adults), and young adults living in the neighboring host community (ages 18–24) to understand their perceptions of the Rohingya and examine if and how the Rohingya may be influencing marriage practices outside the camps. Criterion sampling was used to select study participants. Participants were selected from household listings if they met selection criteria for either in-depth interview or focus groups discussions (age, sex, marital status, position) and were willing to consent to participation in the research. Community and religious leaders were selected based on their positions and leadership roles. Service providers were identified using both listings of available resources of agencies working in the camps and recommendations from community leaders. Table 1 provides more information on participant groups.

**Table 1:** Sample size by data collection method

In-Depth Interviews			Focus Group Discussions		
	Rohingya	Program managers and service providers	Community leaders	Rohingya women	Host community members
Sample (n)	48	24	4 groups of 10–12 in each group	2 groups of 10–12 in each group	6 groups of 10–12 in each group
Population	Males and females, unmarried and married	NGOs, INGOs, UN, DGHS, doctors, paramedics and frontline workers	Mahjees^a^ and Imams	Adult females	Male and female young adults

^a^Mahjees are community leaders or representatives of the Rohingya people. They are appointed by Bangladesh authorities for maintaining control and order inside the camps and work as the focal person for camp management activities including aid distribution and communicating messages from Bangladesh authorities

**Fig. 1:**
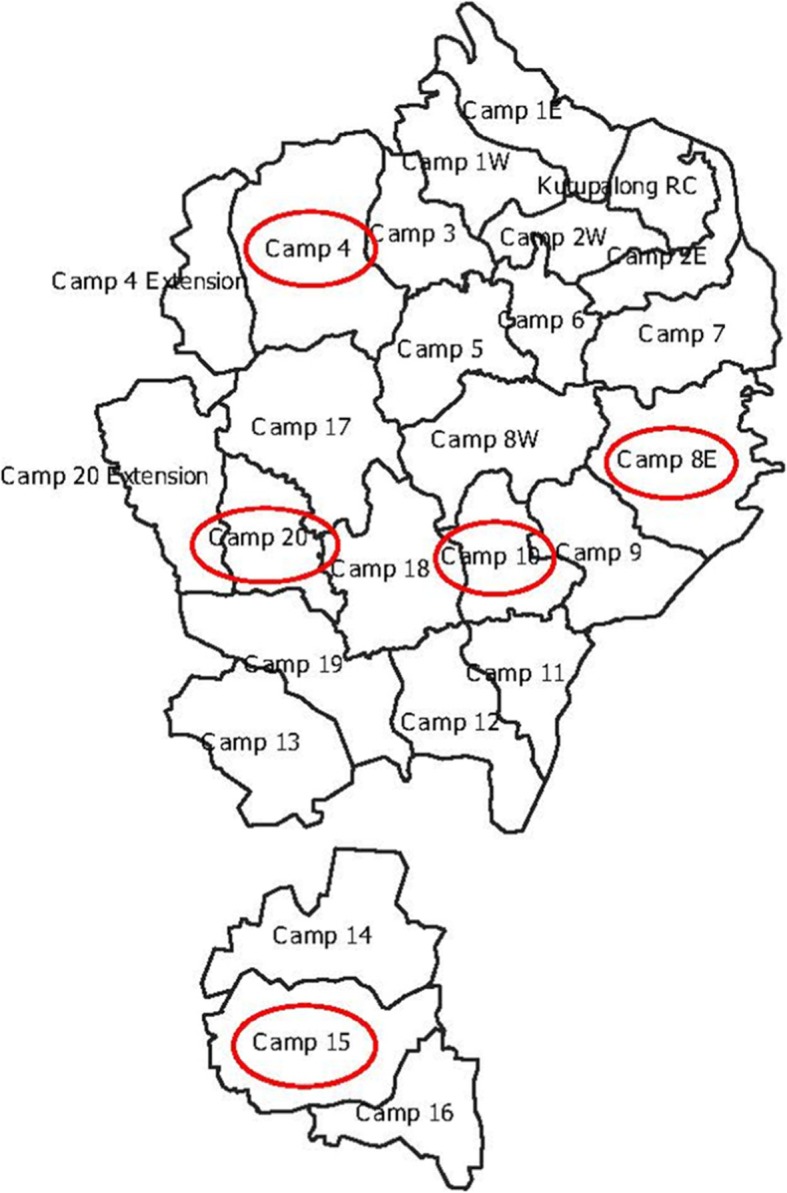
Study Sites This figure shows the Rohingya camps in Bangladesh with the red circles indicating locations of data collection for this particular study. Spatial files were provided by Humanitarian Data Exchange v1.33.1, a service provided by the United Nations Office for the Coordination of Humanitarian Affairs, and are covered under a Creative Commons Attribution 4.0 International license

In-depth interviews and focus group discussions were conducted in July and August 2018 in study sites as shown in Fig. 1. Data were collected from Balukhali- Kutupalong expansion site and the surrounding host community of Palongkhali Union of Ukhia upazila. Five camps (out of 20) were purposively selected to achieve geographic diversity within the camps (East, West, South, North and Center) of the larger camp area as shown in Fig. 1. Table 2 shows the population size of each of the selected camps [50].

**Table 2:** Population of data collection sites in Palong Khali Union, Ukhia upazila

Camp	Total households	Total population
Camp 4	7981	32,115
Camp 8E	7202	31,216
Camp 10	7649	32,963
Camp 15	11,182	49,443
Camp 20	1770	7326

Source: [50]

Research assistants (RAs) were selected based on their ability to converse in *Chatgaya* or the Chittagonian dialect, which shares 70% similarity with the Rakhine language [45].^1^ During data collection training the team developed a glossary of terms across languages that was updated and modified during fieldtesting. More information about that process is available in Ainul et al [3]. Additionally, Rohingya young adults (*n* = 6) from the camps were recruited to assist the RAs during data collection in camps. All of the recruited Rohingya young adults had some experience working with their community and served as insiders with regards to understanding unfamiliar dialects and concepts that arose during IDIs and FGDs.

Given the circumstances surrounding their migration to Bangladesh and their status as stateless, conducting ethical research and protecting participants and Rohingya youth assistants was of the utmost concern. Consent (adults) and permission and assent (minors) were acquired, and we took care to ensure confidentiality and respect were maintained at all times by the research team, and referral provisions were made for additional counseling or services as needed. The research protocol was approved by the Population Council Institutional Review Board.

Interviews and focus group discussions were recorded and transcribed into Bengali from the Chatgaya dialect and then translated into English. Transcripts were reviewed and checked for accuracy and completeness. We maintained full transcripts of the same IDIs and FGDs in both English and Bengali. Data analysis used a thematic approach: three senior researchers from the Dhaka office read the same transcripts in Bengali and coded individually, using English codes. The codes were then compared and summarized to generate a common codebook including themes identified in the instruments and new themes that emerged during initial transcript review. Senior researchers from the U.S. coded transcripts in English using the codebook developed by Dhaka-based researchers [43] was used to manage coding across the two languages and allow for sharing of memos.

Initial analyses as part of a larger study focused on themes related to health services, understanding the sexual and reproductive health needs of the Rohingya living in camps, and marriage practices. Findings from that study are presented elsewhere [3]. This analysis focused on whether and how age at marriage among the Rohingya is influenced by living in the camps in Bangladesh versus living in Myanmar. We used sub codes under marriage practices to examine readiness for and timing of marriage, partner selection, and marriage processes including payment of dowry, marriage registration, and marriage ceremonies. We coded data referring to life in the camps versus in Myanmar to compare differences. Descriptors were linked to transcripts to examine topics across sub-groups.

## Results

### Marriage under military rule: marriage practices in Myanmar

In-depth interviews revealed that marriage practices among the Rohingya were more highly regulated in Myanmar than in the camps. We heard about the broad-ranging influence of the military in their lives prior to the camps: the military played a significant role in restricting mobility, economic participation, access to health care services and education, and social practices including marriage. Respondents spoke of the role of the military in enforcing rules about child marriage, suggesting that living under military rule made child marriages less common as military enforcement prevented some marriages from occurring:

*You could not get married before the age of 18. And if anyone married in secret and the government found out about it, they beat them up and fined them heavily. Getting married there was a very difficult matter.*

-IDI 29, unmarried girl

This resulted in reportedly less child marriage in Myanmar as a survival strategy for the Rohingya, though some respondents suggested that the military’s involvement in child marriages was less about enforcement of rules and more about collecting money from the Rohingya in the form of fines (for breaking the marriage law). As one respondent noted, the military exerted control over age at marriage and marriage ceremonies:

*It was very difficult to get married back there since marriage before the age of 18 was not permitted. There was a military law … Also, the groom’s family and friends could not come over for a ceremony. Loudspeakers could not be used; if the military heard it, they would seize everything. Moreover, you had to pay them for the marriage.*

-IDI 2, unmarried girl

In contrast to reports of military presence delaying marriage, some respondents reported that child marriage was preferable in Myanmar, due in part to wanting to protect daughters. Marriage served as a perceived protective strategy to ensure sexual security, family honor, and fertility. Marriage was seen as worth the associated bribe to allow the marriage to take place if you could afford it:

*In the case of girls, if they are pretty then the boys bother them. Also most of the time there is the fear of military abduction if she catches their eye. That is why parents rush to get their daughters married. If they are wealthy enough, they pay the military a bribe so they can arrange a child marriage.*

-IDI 5, unmarried girl

During the period of reduced Nasaka armed presence in the region some respondents noted that many girls got married at an early age. It is not clear whether this was due to preferring child marriage as a practice, or as an economic strategy to avoid the fines or bribes associated with child marriage, or some combination:

*For a while the Nasaka had left. When they were gone, it was no longer required to deposit any money for marriages and even permission was not required. All the boys and girls got married then. At that time some girls were married off within a year of their first period or less.*

-IDI 2, unmarried girl from camp 10

Although overall the majority of respondents reported that marriage was more highly regulated in Myanmar than in the camps, how marriage practices unfolded in the absence of the Nasaka may demonstrate marriage preferences among the Rohingya. This may be an important indicator of how marriage practices in the camps unfold. Although we heard that life in the camps is restricted in different ways, it is reportedly less restricted than living under military rule and the Rohingya have more freedom in marriage timing and practices.

### Relaxing of laws: marriage practices in the camps

Participants suggest that living in the camps influences the age at which girls get married with girls being married at younger ages than they would be married in Myanmar. As one unmarried girl told us, the role of the military in Myanmar used to mean that rules against marriage before age 18 were enforced. However, now living in the camps, there is less enforcement and, based on her perception, more child marriage. She notes:

*Previously, people used to get married after becoming 18 years old in our society. But at present, people don’t wait until 18 years of age. They don’t hesitate to marry even if they are younger than 18 years. People are getting their children married at any age- be it at 18 years, 15 years or even 10 years of age. The fact is, whenever people are getting suitable proposals, they are getting their children married. They are not looking at the age. Girls have to get married as soon as their menstrual cycle starts, and achieve noticeable physical change around puberty. That may happen at 15 years, or even 12 years of age. Similarly, if boys also achieve noticeable physical changes around puberty, they have to get married soon. Some boys even get married before 18 years of age.*

-IDI 14, unmarried girl

A young married girl noted the change in marriage practices from military rule in Myanmar to practices in the camps in Bangladesh:

*In our community you could say there were almost no child marriages [in Myanmar]. The laws were very strict. It was very important to follow the military rules. But after coming to the camp, child marriage has become a regular thing. A lot of girls are getting married before the age of 18. There are no specific rules here.*

-IDI 4, married girl

In the absence of military regulations around marriage, community members now play a lead role in marriage practices. Participants noted that Mahjees, unelected refugee leaders, [49] have administrative duties that include reporting marriages and preparing marriage contracts:.

*The Mahjees inform the armies in charge of the camp. The Mahjee also prepare the marriage contracts, the Head Mahjee does this.*

- IDI 15, unmarried girl

Marriage practices in the camps may reflect group preferences for child marriage that existed in Myanmar but were not easily acted upon. We heard from some Rohingya participants that preferences around age at marriage are rooted in Islam. As one unmarried girl noted-

*“Girls have to get married as soon as their menstrual cycle starts because it is a religious obligation in Islam for parents to get their children married.”*

-IDI 14, unmarried Rohingya girl.

Others echoed the same sentiment that religion plays a major role in dictating the timing of marriage and discussed how it was different back in Myanmar:

*Our religion says that when a boy grows up and understands the meaning of a wife [with regards to religion], he should get married. If the girl grows up, she should get married. This is told in Islam. We couldn’t abide by those beliefs back there. We couldn’t follow this rule in fear of the Mogs (slang for Myanmar army) and the government. We came here and now people can follow these rules. However, this was not allowed in Myanmar. They [Burmese] could do it but we were not allowed. If we said that we want to do it, they asked us to pay more money for that. Where would we get so much money? Still we did it secretly. They used to beat people if they were caught, then took more money as a fine.*

-IDI 31, married Rohingya male

As this respondent notes, the Rohingya in Myanmar preferred early marriage due in part to religious beliefs, but acting upon these preferences were difficult and marriages sometimes occurred in secret or only after bribes were paid.

### The changing cost of marriage

One reason why marriage practices may have changed since leaving Myanmar is that the costs associated with marriage have changed for the Rohingya. These changes are due to less access to funds because of restrictions around work in the camps, fewer fines and bribes to pay than back in Myanmar, and reportedly lower dowry costs.^2^ By virtue of their legal status and lack of livelihood opportunities, Rohingya do not have the funds to pay for marriage in the way they might have back in Myanmar. However, marriage costs are also lower in the camps since fees and bribes expected for marriage in Myanmar are not required in the camps in Bangladesh. Some respondents felt the changing costs of marriage presents an opportunity to marry that may not have been available in Myanmar. Girls from more economically disadvantaged families may be better able to enter the marriage market in the camps than they were back in Myanmar:

*I have seen a new system running in the camps. Previously 35 lac-50 lac kyat [2300 to 3300 USD] was paid as dowry at the time of marriage, but now even 2 thousand [22 USD] or 1 thousand [11 USD] kyat is sufficient as a dowry payment. Grooms’ families are taking the brides home without any dowry but with love and affection. It is even seen that the girl who couldn’t get married back in Myanmar due to financial limitations, got married after coming here.*

-Focus group with Mahjees

The costs associated with marriage are also lower in the camps because there are fewer authorities to satisfy before formalizing a marriage. In Myanmar, regulation of marriage by requiring payments including bribes, fines, and fees in order to marry served to restrict child marriage. Now in the camps, some of those restrictions are absent and this brings a new freedom for the Rohingya to exercise choice in when to marry. In some cases, this means marriage happens before age 18:

*Yes, I’ve seen a wedding in this camp. The bride was 15–16 years old. And the groom was 18 years old. The contract was prepared in presence of the community leaders.*

-IDI 14, unmarried girl

However, despite lower costs attached to marriage, the Rohingya have fewer resources so they may not be able to afford marriage even with reduced associated costs. As one man noted here, money may still be a barrier to marriage:

*It was very hard to get girls married in Myanmar, because you had to pay money. Also there were many girls who could not get married due to the lack of money, so they remained in their homes. There are also many girls who could not get married even after coming here because of money.*

-IDI 35, married man

As the Rohingya have few options for livelihoods in the camps, the marriage market may not be able to support the levels of dowry once demanded. Although some Rohingya reported bringing gold and other assets with them from Myanmar, many reported having nothing of value.

*Since no one has much wealth here dowry is not a necessity here. Still there are some who are getting married in exchange for dowry. And unlike before not much paper work is needed here. And also we don’t have to pay the military like before. People are getting married with whatever little capability they have.*

-IDI 18, unmarried girl

When payments do occur in the camps, some reported that these payments are less than they would have been back in Myanmar, though whether this is due to an overall reduced cost because of reduced ability to afford marriages or reduced costs in response to increased supply (younger girls now in the marriage market) is not clear.

*The ritual here is that girls don’t have to pay anything. They had to if they were in Myanmar. The takers [Myanmar officials] would take more money, many people had to pay more. Boys like me and my friends got married,[but] they had to pay two hundred lakhs, one hundred lakhs.*

*Here, if someone is giving money then they take it. People agree with what is given from their own will, and not much is needed.*

-IDI 27, married man

### Preferences for age at marriage among the Rohingya

Both Rohingya girls and adults in the camps in Bangladesh report a preference for child marriage in their community. As one unmarried 18 year old girl told us, earlier is better when it comes to marriage:

*In our society, the sooner people can get their daughters married, the better. If a girl can’t get married early, her eligibility diminishes with time. Therefore, the parents try to get their daughters married before it’s too late, or the girls become too old to get married. Age is not a problem in the case of boys, but it is a problem for girls. It is a big problem if a girl can’t get married before she becomes 18 years old. Then nobody wants to marry her. She is considered too old by society. Her eligibility diminishes as her fertility also decreases with time. That’s why girls get married as early as possible in our society. No one marries girls who have become 18 years old. They consider them too old to get married.*

-IDI 14, unmarried girl

Recognizing that preferences for age at marriage can reflect both individual preferences as well as social norms regarding marriage, we sought to understand the markers that signal readiness for marriage among the Rohingya and the family and social forces that influence age at marriage. We find that family need and economic burden are important factors driving preference for child marriage. One married girl spoke of family size and needing to alleviate burden on a family by getting married:

*In the Rohingya society in Myanmar, the number of girls is higher. Each family has 4–6 girls. Thus, the house is filled with girls. If they keep their girls at home, no one wants to marry them saying that they have become too old. People say ‘the girl has become old’, thus they get their daughters married before they get old. Besides that, no one wants to keep three/four young girls home at the same time. People like us who don’t have money try to get their daughters married soon with the money they can manage.*

-IDI 12, married girl

In addition to alleviating family burden, there are physical markers that denote a transition to marital readiness. As in other countries where child marriage is high, menstruation marks readiness for adult responsibilities [16, 33], including marriage. Religion also suggests that physical maturity marks readiness for marriage as an earlier quote noted: *if the girl grows up, she should get married, this is told in Islam (*IDI 31, married Rohingya male). Physical changes at puberty including menstruation may also serve as a warning that there is limited time to secure a girl’s safety through marriage as males in the community may begin to see her as a sexual being. This raises concerns related to a fear of sexual abuse and fear of reputational risk if the girl engages in premarital sex.

*When there are 3–4 daughters in a family, the eldest one has to get married as soon as her menstrual cycle starts, so that the immediately younger one can also get married off in the same way. This is done to save the reputation of the family, and to keep her from committing sins. Many girls commit sins before getting married. These can be prevented if they are married off as early as possible.*

-IDI 15, unmarried girl

As others have noted, concerns about sexual violence and control of adolescent sexuality are important factors driving child marriage across contexts [18], including among Rohingya participants in this research.

### Effect of age at marriage in the camps on the host community

In general, interviews with host community members revealed tension between Bangaldeshis and the Rohingya. Interviews with individuals in the host community covered a number of topics including their impressions about the influence of the Rohingya on economic, health, environmental, and social aspects of their lives as host community members. There were many negative associations with the Rohingya, including statements such as *“They should be sent off to their country. Two different communities can never stay together”* (FGDs with unmarried girls in host community). We also heard concerns about environmental degradation and deforestation as well as security concerns that have reportedly increased since the influx [3].

We were specifically interested in understanding how the host community views the timing of marriage and whether child marriage (observed) or perceptions of child marriage in the camps influence practices outside of the camps as well. Despite hearing from Rohingya girls that marriages to non-Rohingya men do not occur, participants from a focus group of unmarried girls in the host community reported that they do occur, and that the presence of Rohingya women and girls in the camps may also influence polygamy among Bangladeshi men:

*Both married and unmarried Bangladeshi men are marrying Rohingya girls... The husband of a girl in our area brought a Rohingya girl here after marrying her. That Rohingya girl is a teacher at [name redacted for confidentiality] school … Five boys from this village and even their fathers have married Rohingya girls after the recent influx. Fights are happening in families due to this. There are now two wives in each family. All these Rohingya girls are unmarried. They are marrying Bangladeshi men who are as old as their fathers and grandfathers. This disrupts the peace within a family.*

-FGD 5, unmarried girls in host community

In addition to what we heard about the presence of Rohingya women and girls directly influencing marriage practices, we were also interested to understand how perceptions of the normative age at marriage among the Rohingya may influence the host community. We examined host community and service provider perceptions of age at marriage in the camps to understand how assumptions of what is normative in the camps may influence behavior outside of the camps. Service providers also reported that they perceive child marriage to be common among the Rohingya. However, some service providers estimated the age at marriage among the Rohingya to be even younger than what was reported in interviews by the Rohingya directly.

*In their community, girls are married off at 12–13 years of age. Fourteen to fifteen year old girls who are yet to be married off are rarely found here. Boys generally marry at 16–17 years of age.*

-IDI 7, male NGO worker

However, when asked about witnessing any marriages in the camps, this respondent had not:

*R: As my workstation is in Cox’s Bazar, I haven’t seen any weddings take place in the camps, but heard of those from other staff who are working in camps.*

*Q: Which type of people are the boys and girls of the camps getting married to?*

*R: They are getting married within their community. I haven’t so far heard of any marriages taking place outside of the Rohingya community.*

-IDI 7, male NGO worker

This service provider had not witnessed any child marriages directly, but other service providers did report treating women who had reportedly been married as children.

*During my work tenure at the camp, I have seen many married women with so many kids. They got married at an early age which we call child marriage. Child marriage can create many sexual and reproductive health risks for women.*

-IDI 51, service provider, midwifery

Although service providers may be different from other host community members, their input provides an important example of what others think is normative with regards to the Rohingya.

## Discussion

Our findings suggest that preferences for marriage under age 18 among the Rohingya are more easily expressed in the camps in Bangladesh than they were in Myanmar. This preference was likely present in Myanmar but restrictions prevented most marriages from occurring at the preferred time. Now we may see more child marriages happening in the camps as compared to Myanmar as restrictions have loosened. The absence of restrictions on age at marriage in the camps combined with vulnerabilities to child marriage including economic insecurity, refugee status, and climate effects [4, 28, 35]. make it that girls will continue to marry early in the camps.

Important contextual changes after the Rohingya were displaced from Myanmar may help to explain what appears to be an increase in child marriage in the camps in Bangladesh. Participants suggested these preferences are strongly influenced by religious norms around the timing of marriage. While participants told us that Islam dictates marriage once girls reach physical maturity, other research has noted that although Islam is used to justify practices like child marriage and forced genital mutilation/cutting (FGM/C), not all Islamic countries have a high prevalence of these practices. Additionally, non-Islamic countries also grapple with child marriage and FGM/C, suggesting that religion, particularly Muslim identity, is not a sufficient explanation for preferences for child marriage [52]. Rather than simply reflecting religious preference some combination of culture, religion, poverty, political vulnerability, and group norms are likely a better explanation for why the Rohingya may demonstrate a preference for child marriage. Research by Leider [26] suggests that the Rohingya in Myanmar identified more strongly with religion as a unifying characteristic than ethnicity [26]. This identification with religion as a unifying characteristic may in part explain why Islam is frequently cited by participants in this research as a determination in decisions about timing of marriage.

How participants talk about the cost of marriage also suggests that economic aspects of marriage are an important consideration in age at marriage among the Rohingya living in the camps in Bangladesh. Marriage is a transaction that responds to market changes: among the Rohingya in Bangladesh we hear reported differences in expectations about dowry (less payments required) and overall costs (bribes no longer necessary) that reduce financial barriers and open the market to girls who may have had limited marital opportunities in Myanmar. However, at the same time the Rohingya in Bangladesh have limited financial means that effect ability to afford marriages as they may have in Myanmar due to increased restrictions on work in the camps.

Beyond preferences for child marriage, our research aligns with findings from studies that note that insecurity, whether related to climate or conflict, increases gender inequities including child marriage. Displacement and insecurity increase financial pressures and child marriage may alleviate some of the immediate economic burden on families. Marriage may also serve as a means to keep girls safe from sexual abuse and reputational threats related to female sexuality that influence a girl’s marriage prospects.

There are a few limitations to note. Although we speculate based on qualitative data that child marriage is more common in the camps than it was in Myanmar, we do not have quantitative data to understand whether prevalence of child marriage has increased among the Rohingya after displacement to Bangaldesh. An additional limitation is that due in part to their status as refugees living in the camps, responses may be more influenced by social desirability in this group than among respondents that are not displaced persons in a precarious legal situation. Additionally, as NGO-affiliated research staff, interviewers may have been seen as gatekeepers to important services as the Rohingya camps are the beneficiaries of NGO-implemented aid for basic health services. Thus, NGO-affiliation could be seen as a direct link to aid and this could influence responses. We included language in consent forms to mitigate this but we acknowledge it may still influence some responses. Additionally, although we took great care to ensure that language issues did not hinder our understanding of these topics [3] there is chance that some language issues remained.

It is unlikely that we will see a decline in child marriages in the camps any time soon. Rather, it is likely that child marriages will continue among the Rohingya in the camps. This is a concern for those living in the camps, as we know child marriage is associated with a number of negative outcomes for girls, but may also have a negative effect on the host population. It is currently unclear whether normalizing child marriage in the camps will extend beyond the camps to the neighboring host community, but over time it is likely to have a notable social or market influence on age at marriage or practices around polygamy. For example, some host community respondents spoke of the influence of the Rohingya already on marriage in the host community, with some reports of Bangladeshi men taking Rohingya girls as second wives. Young marriageable girls may also influence the marriage market in host communities as dowry may be lower for these women at younger ages, but rise as they get older. This may incentivize parents to facilitate marriages earlier, especially in families with multiple daughters. Dowry payments are still common in Bangladesh despite being illegal with some reports that they are becoming more common [4]. Host communities may also be influenced by seeing what is normative in the camps with regards to age at marriage and this may influence the timing of marriage for girls in neighboring communities outside of the camps. It is important to note that host community members may also be influenced by media messaging about child marriage within Bangladesh. Despite a significant decline in child marriage in Bangladesh overall (from 73.0% of women 20–24 married by age 18 in 1993/4 to 59.0% in 2014) it still remains a significant problem and receives media attention, particular related to a much debated adjustment to the law in 2017 [10]. More research is needed to understand how marriage practices in both the Rohingya camps and in the host community influence one another and what strategies may be most effective to counter trends in child marriage across both settings.

Given the status of statelessness and the low likelihood that the Rohingya will be given refugee status, legal enforcement of age of marriage laws is unlikely in the camps. There is considerable reluctance to introduce access to justice initiatives for the Rohingya for anything but criminal cases. Even if laws were enacted and enforced, in the absence of birth registration and age documentation enforcement mechanisms would be weak. Reports suggest that the camps themselves encourage child marriage as food rations are distributed by household and marriages entail the creation of new households [29]. Although we did not hear this from respondents, it is worth considering how aid structures may contribute to child marriage. It is conceivable that the preference for child marriage in the camps could respond to interventions to enhance girls’ capabilities as shown in successful programs to delay marriage using livelihoods and educational opportunities [6, 7] and education [19] as alternatives to marriage. However, programs would need to work around limitations in the camps and there are limited examples of successful empowerment programs for girls in refugee settings.

## Conclusions

These data highlight that there is a preference for child marriage among the Rohingya and this preference is more easily expressed (and acted upon) in the camps in Bangladesh as compared to Myanmar. The practice of child marriage may have consequences for both the Rohingya population living in the camps as well as the neighboring host community. The findings presented here suggest that while these preferences are presented as motivated by cultural factors or justified by religion, there are strong underpinnings in terms of financial considerations and concerns about girls’ safety. Humanitarian agencies have long recognized the importance of ensuring security and safety. Interventions that contribute to safety for girls can have important implications for marriage practices. In addition, safe spaces programs that include skill building to build resilience may be more feasible and sustainable than enforcement of laws to reduce child marriage in camp setting, both for the Rohingya population and host community.

## Data Availability

The qualitative datasets generated and analyzed during the current study are not publicly available but are available from the corresponding author on reasonable request.

## Abbreviations

DGHS: Directorate General of Health Services, an agency in the Ministry of Health & Family Welfare
FGD: Focus group discussion
IDI: In-depth interview
INGO: International non-government organization
NGO: Non-government organization
RA: Research Assistants

## Footnotes

1 According to the Translators without Borders (TWB) language assessment held in 2017, Rohingya speakers estimated that there was a 70% similarity between Chittagonian and Rohingya (TWB 2017). It also noted that a 90% similarity was estimated between the Cox’s Bazar dialect of Chittagonian and Rohingya because of the close geographic proximity of these two regions [﻿46﻿]. A glossary is available in [3].

2 A few respondents reported that dowry expectations are higher in the camps but generally speaking most participants reported dowry being lower.
